# Supplementary material for: The Illusion of Simplicity: The Dramatic Failure of Koopmans’ Theorem for Antioxidants in Solvents—The Ascorbic Acid Paradigm
Source: Chemphyschem. 2026 Jul 10;27(13):e70375. doi: 10.1002/cphc.70375 (PMC13354722; doi:10.1002/cphc.70375)
Supplement: Supplementary file 1 — Supplementary Material [file CPHC-27-e70375-s001.pdf]

Supporting Information The Illusion of Simplicity: The Dramatic Failure of Koopmans Theorem for Antioxidants in Solvent — The Ascorbic Acid Paradigm

Ioan Bâldea

ioan.baldea@pci.uni-heidelberg.de

Table S1. Global reactivity descriptors and solvation Gibbs free energies for ascorbic acid in **Benzene**. Reactivity descriptors in eV, ΔG_{sol} in kcal/mol. Upper subrow: absolute values; lower subrow: signed deviation from CBS-QB3.

Method	IP	EA	E_g	η	σ	χ	ω	ω^+	ω^-	ΔG^n_{sol}	ΔG^c_{sol}	ΔG^a_{sol}
**CBS-QB3**	7.273	0.775	6.498	3.249	0.154	4.024	2.491	0.886	4.909	-4.817	-29.084	-30.722
	0.000	0.000	0.000	0.000	0.000	0.000	0.000	0.000	0.000	0.000	0.000	0.000
B3LYP/6-311++G(3df,3pd)	6.980	0.850	6.130	3.065	0.163	3.915	2.500	0.926	4.841	-4.971	-29.709	-27.514
	-0.293	+0.075	-0.368	-0.184	+0.009	-0.109	+0.009	+0.040	-0.069	-0.154	-0.625	+3.208
B3LYP/6-311+G(3df,3pd)	6.980	0.847	6.133	3.067	0.163	3.913	2.497	0.924	4.837	-4.971	-29.711	-30.035
	-0.293	+0.072	-0.365	-0.182	+0.009	-0.110	+0.006	+0.038	-0.072	-0.154	-0.627	+0.687
B3LYP/6-311G(3df,3pd)	6.830	0.549	6.281	3.141	0.159	3.689	2.167	0.715	4.404	-4.434	-29.079	-31.049
	-0.443	-0.226	-0.217	-0.109	+0.005	-0.334	-0.324	-0.171	-0.505	+0.383	+0.005	-0.327
B3LYP/6-31+G(d,p)	6.986	0.917	6.069	3.035	0.165	3.952	2.573	0.976	4.928	-5.475	-30.268	-30.726
	-0.287	+0.142	-0.429	-0.214	+0.011	-0.072	+0.081	+0.090	+0.018	-0.658	-1.184	-0.004
M062X/6-311++G(3df,3pd)	7.131	0.740	6.391	3.195	0.156	3.936	2.424	0.855	4.791	-4.851	-29.686	-31.100
	-0.141	-0.034	-0.107	-0.054	+0.003	-0.088	-0.068	-0.030	-0.118	-0.035	-0.602	-0.378
M062X/6-311+G(3df,3pd)	7.132	0.739	6.393	3.196	0.156	3.935	2.422	0.854	4.789	-4.851	-29.677	-31.145
	-0.141	-0.036	-0.105	-0.053	+0.003	-0.089	-0.069	-0.031	-0.120	-0.035	-0.593	-0.423
M062X/6-311G(3df,3pd)	7.010	0.495	6.515	3.257	0.153	3.753	2.162	0.692	4.445	-4.453	-29.187	-31.148
	-0.263	-0.279	+0.017	+0.008	-0.000	-0.271	-0.330	-0.193	-0.464	+0.363	-0.103	-0.426
M062X/6-31+G(d,p)	7.120	0.801	6.319	3.160	0.158	3.960	2.482	0.897	4.857	-5.450	-30.008	-31.773
	-0.153	+0.026	-0.179	-0.089	+0.004	-0.063	-0.009	+0.011	-0.052	-0.633	-0.924	-1.051

Table S2. Global reactivity descriptors and solvation Gibbs free energies for ascorbic acid in **Toluene**. Reactivity descriptors in eV, ΔG_{sol} in kcal/mol. Upper subrow: absolute values; lower subrow: signed deviation from CBS-QB3.

Method	IP	EA	E_g	η	σ	χ	ω	ω^+	ω^-	ΔG^n_{sol}	ΔG^c_{sol}	ΔG^a_{sol}
**CBS-QB3**	7.183	0.761	6.422	3.211	0.156	3.972	2.456	0.872	4.843	-5.029	-30.184	-31.847
	0.000	0.000	0.000	0.000	0.000	0.000	0.000	0.000	0.000	0.000	0.000	0.000
B3LYP/6-311++G(3df,3pd)	6.889	0.835	6.055	3.027	0.165	3.862	2.463	0.911	4.773	-5.192	-30.889	-28.622
	-0.293	+0.074	-0.367	-0.184	+0.009	-0.109	+0.007	+0.039	-0.070	-0.163	-0.705	+3.225
B3LYP/6-311+G(3df,3pd)	6.889	0.832	6.057	3.029	0.165	3.861	2.461	0.909	4.770	-5.193	-30.887	-31.143
	-0.293	+0.071	-0.365	-0.182	+0.009	-0.111	+0.005	+0.037	-0.074	-0.163	-0.703	+0.704
B3LYP/6-311G(3df,3pd)	6.740	0.534	6.206	3.103	0.161	3.637	2.132	0.701	4.338	-4.634	-30.165	-32.002
	-0.443	-0.226	-0.216	-0.108	+0.005	-0.335	-0.325	-0.171	-0.505	+0.395	+0.019	-0.155
B3LYP/6-31+G(d,p)	6.896	0.902	5.994	2.997	0.167	3.899	2.537	0.962	4.861	-5.722	-31.369	-31.874
	-0.286	+0.142	-0.428	-0.214	+0.011	-0.072	+0.081	+0.090	+0.018	-0.692	-1.185	-0.027
M062X/6-311++G(3df,3pd)	7.041	0.727	6.314	3.157	0.158	3.884	2.389	0.842	4.726	-5.073	-30.780	-32.240
	-0.142	-0.034	-0.108	-0.054	+0.003	-0.088	-0.067	-0.030	-0.118	-0.043	-0.596	-0.393
M062X/6-311+G(3df,3pd)	7.041	0.725	6.316	3.158	0.158	3.883	2.387	0.841	4.724	-5.073	-30.771	-32.287
	-0.142	-0.035	-0.106	-0.053	+0.003	-0.088	-0.069	-0.031	-0.120	-0.043	-0.587	-0.440
M062X/6-311G(3df,3pd)	6.920	0.482	6.438	3.219	0.155	3.701	2.128	0.680	4.381	-4.657	-30.233	-32.283
	-0.263	-0.278	+0.016	+0.008	-0.000	-0.270	-0.328	-0.192	-0.463	+0.373	-0.049	-0.436
M062X/6-31+G(d,p)	7.030	0.787	6.243	3.121	0.160	3.909	2.447	0.883	4.792	-5.697	-31.150	-32.947
	-0.153	+0.027	-0.179	-0.090	+0.004	-0.063	-0.009	+0.011	-0.051	-0.668	-0.966	-1.100

Table S3. Global reactivity descriptors and solvation Gibbs free energies for ascorbic acid in **Chlorobenzene**. Reactivity descriptors in eV, ΔG_{sol} in kcal/mol. Upper subrow: absolute values; lower subrow: signed deviation from CBS-QB3.

Method	IP	EA	E_g	η	σ	χ	ω	ω^+	ω^-	ΔG^n_{sol}	ΔG^c_{sol}	ΔG^a_{sol}
**CBS-QB3**	5.886	0.443	5.444	2.722	0.184	3.164	1.840	0.598	3.762	-8.190	-43.569	-47.225
	0.000	0.000	0.000	0.000	0.000	0.000	0.000	0.000	0.000	0.000	0.000	0.000
B3LYP/6-311++G(3df,3pd)	5.586	0.506	5.080	2.540	0.197	3.046	1.827	0.621	3.667	-8.507	-44.598	-43.830
	-0.300	+0.064	-0.364	-0.182	+0.013	-0.118	-0.013	+0.024	-0.095	-0.317	-1.029	+3.395
B3LYP/6-311+G(3df,3pd)	5.586	0.505	5.081	2.541	0.197	3.046	1.825	0.620	3.666	-8.510	-44.601	-46.379
	-0.300	+0.062	-0.363	-0.181	+0.013	-0.119	-0.014	+0.023	-0.096	-0.319	-1.032	+0.846
B3LYP/6-311G(3df,3pd)	5.435	0.214	5.221	2.610	0.192	2.824	1.528	0.442	3.266	-7.598	-43.563	-46.679
	-0.452	-0.229	-0.223	-0.111	+0.008	-0.340	-0.312	-0.156	-0.496	+0.592	+0.006	+0.546
B3LYP/6-31+G(d,p)	5.603	0.576	5.027	2.514	0.199	3.089	1.899	0.668	3.757	-9.446	-45.119	-47.550
	-0.283	+0.133	-0.416	-0.208	+0.015	-0.075	+0.059	+0.070	-0.005	-1.256	-1.550	-0.325
M062X/6-311++G(3df,3pd)	5.748	0.415	5.332	2.666	0.188	3.081	1.781	0.573	3.655	-8.370	-44.191	-47.847
	-0.139	-0.027	-0.111	-0.056	+0.004	-0.083	-0.059	-0.024	-0.107	-0.180	-0.622	-0.622
M062X/6-311+G(3df,3pd)	5.748	0.414	5.333	2.667	0.187	3.081	1.780	0.573	3.654	-8.372	-44.194	-47.916
	-0.139	-0.028	-0.110	-0.055	+0.004	-0.083	-0.060	-0.025	-0.108	-0.181	-0.625	-0.691
M062X/6-311G(3df,3pd)	5.628	0.178	5.450	2.725	0.183	2.903	1.546	0.435	3.338	-7.644	-43.206	-47.710
	-0.258	-0.265	+0.007	+0.003	-0.000	-0.262	-0.293	-0.162	-0.424	+0.547	+0.363	-0.485
M062X/6-31+G(d,p)	5.743	0.479	5.264	2.632	0.190	3.111	1.839	0.612	3.723	-9.355	-44.727	-49.097
	-0.143	+0.036	-0.180	-0.090	+0.006	-0.054	-0.001	+0.015	-0.039	-1.165	-1.158	-1.872

Table S4. Global reactivity descriptors and solvation Gibbs free energies for ascorbic acid in **Methanol**. Reactivity descriptors in eV, ΔG_{sol} in kcal/mol. Upper subrow: absolute values; lower subrow: signed deviation from CBS-QB3.

Method	IP	EA	E_g	η	σ	χ	ω	ω^+	ω^-	ΔG^n_{sol}	ΔG^c_{sol}	ΔG^a_{sol}
**CBS-QB3**	5.835	0.999	4.836	2.418	0.207	3.417	2.414	1.008	4.424	-10.594	-53.353	-57.058
	0.000	0.000	0.000	0.000	0.000	0.000	0.000	0.000	0.000	0.000	0.000	0.000
B3LYP/6-311++G(3df,3pd)	5.533	1.057	4.476	2.238	0.223	3.295	2.426	1.058	4.353	-11.051	-54.042	-53.803
	-0.302	+0.059	-0.360	-0.180	+0.017	-0.121	+0.012	+0.050	-0.071	-0.457	-0.689	+3.255
B3LYP/6-311+G(3df,3pd)	5.533	1.056	4.477	2.239	0.223	3.295	2.425	1.057	4.352	-11.054	-54.044	-56.370
	-0.301	+0.058	-0.359	-0.180	+0.017	-0.122	+0.011	+0.049	-0.072	-0.460	-0.691	+0.688
B3LYP/6-311G(3df,3pd)	5.385	0.771	4.615	2.307	0.217	3.078	2.053	0.802	3.881	-9.831	-52.738	-56.454
	-0.450	-0.228	-0.222	-0.111	+0.010	-0.339	-0.361	-0.205	-0.544	+0.763	+0.615	+0.604
B3LYP/6-31+G(d,p)	5.551	1.126	4.425	2.212	0.226	3.338	2.519	1.126	4.464	-12.329	-54.952	-57.802
	-0.284	+0.127	-0.411	-0.206	+0.019	-0.078	+0.105	+0.118	+0.040	-1.735	-1.599	-0.744
M062X/6-311++G(3df,3pd)	5.686	0.976	4.710	2.355	0.212	3.331	2.356	0.985	4.316	-10.857	-53.983	-57.927
	-0.149	-0.022	-0.127	-0.063	+0.006	-0.086	-0.058	-0.023	-0.108	-0.263	-0.630	-0.869
M062X/6-311+G(3df,3pd)	5.686	0.976	4.710	2.355	0.212	3.331	2.355	0.984	4.315	-10.856	-53.985	-58.021
	-0.149	-0.023	-0.126	-0.063	+0.006	-0.086	-0.058	-0.023	-0.109	-0.262	-0.632	-0.963
M062X/6-311G(3df,3pd)	5.567	0.744	4.823	2.412	0.207	3.156	2.064	0.788	3.944	-9.888	-52.873	-57.738
	-0.268	-0.255	-0.013	-0.006	+0.001	-0.261	-0.349	-0.220	-0.481	+0.706	+0.480	-0.680
M062X/6-31+G(d,p)	5.680	1.040	4.640	2.320	0.216	3.360	2.433	1.043	4.403	-12.102	-55.014	-59.414
	-0.154	+0.042	-0.196	-0.098	+0.009	-0.056	+0.019	+0.035	-0.021	-1.508	-1.661	-2.356

Table S5. Global reactivity descriptors and solvation Gibbs free energies for ascorbic acid in **Ethanol**. Reactivity descriptors in eV, ΔG_{sol} in kcal/mol. Upper subrow: absolute values; lower subrow: signed deviation from CBS-QB3.

Method	IP	EA	E_g	η	σ	χ	ω	ω^+	ω^-	ΔG^n_{sol}	ΔG^c_{sol}	ΔG^a_{sol}
**CBS-QB3**	5.917	1.040	4.877	2.439	0.205	3.479	2.481	1.047	4.525	-10.417	-52.738	-56.384
	0.000	0.000	0.000	0.000	0.000	0.000	0.000	0.000	0.000	0.000	0.000	0.000
B3LYP/6-311++G(3df,3pd)	5.616	1.099	4.517	2.258	0.221	3.357	2.496	1.099	4.457	-10.858	-53.403	-53.121
	-0.301	+0.059	-0.360	-0.180	+0.016	-0.121	+0.015	+0.053	-0.069	-0.441	-0.665	+3.263
B3LYP/6-311+G(3df,3pd)	5.616	1.098	4.518	2.259	0.221	3.357	2.495	1.099	4.456	-10.860	-53.404	-55.686
	-0.301	+0.058	-0.359	-0.180	+0.016	-0.121	+0.014	+0.052	-0.070	-0.443	-0.666	+0.698
B3LYP/6-311G(3df,3pd)	5.468	0.812	4.656	2.328	0.215	3.140	2.118	0.839	3.979	-9.665	-52.134	-55.776
	-0.449	-0.228	-0.222	-0.111	+0.010	-0.339	-0.363	-0.208	-0.547	+0.752	+0.604	+0.608
B3LYP/6-31+G(d,p)	5.633	1.168	4.465	2.232	0.224	3.400	2.589	1.168	4.569	-12.116	-54.317	-57.102
	-0.284	+0.128	-0.412	-0.206	+0.019	-0.078	+0.108	+0.122	+0.044	-1.699	-1.579	-0.718
M062X/6-311++G(3df,3pd)	5.768	1.017	4.750	2.375	0.211	3.392	2.423	1.023	4.416	-10.679	-53.388	-57.240
	-0.149	-0.023	-0.127	-0.063	+0.005	-0.086	-0.058	-0.023	-0.109	-0.262	-0.650	-0.856
M062X/6-311+G(3df,3pd)	5.768	1.017	4.751	2.376	0.210	3.392	2.422	1.023	4.415	-10.680	-53.390	-57.333
	-0.149	-0.023	-0.126	-0.063	+0.005	-0.086	-0.059	-0.024	-0.110	-0.263	-0.652	-0.949
M062X/6-311G(3df,3pd)	5.649	0.785	4.864	2.432	0.206	3.217	2.127	0.823	4.040	-9.723	-52.304	-57.055
	-0.268	-0.255	-0.013	-0.006	+0.001	-0.262	-0.354	-0.224	-0.485	+0.694	+0.434	-0.671
M062X/6-31+G(d,p)	5.762	1.081	4.681	2.340	0.214	3.422	2.501	1.083	4.504	-11.898	-54.408	-58.706
	-0.155	+0.041	-0.196	-0.098	+0.009	-0.057	+0.020	+0.036	-0.021	-1.481	-1.670	-2.322

Table S6. Mean absolute deviation (MAD, eV) and maximum absolute deviation (MaxAD, eV) for adiabatic ionization potential (IP) relative to CBS-QB3 (averaged over all solvents).

Method	MAD	MaxAD
B3LYP/6-311++G(3df,3pd)	0.296	0.302
B3LYP/6-311+G(3df,3pd)	0.296	0.302
B3LYP/6-311G(3df,3pd)	0.446	0.452
B3LYP/6-31+G(d,p)	0.284	0.287
M062X/6-311++G(3df,3pd)	0.144	0.149
M062X/6-311+G(3df,3pd)	0.144	0.149
M062X/6-311G(3df,3pd)	0.264	0.268
M062X/6-31+G(d,p)	0.152	0.156

Table S7. Mean absolute deviation (MAD, eV) and maximum absolute deviation (MaxAD, eV) for adiabatic electron affinity (EA) relative to CBS-QB3 (averaged over all solvents).

Method	MAD	MaxAD
B3LYP/6-311++G(3df,3pd)	0.090	0.242
B3LYP/6-311+G(3df,3pd)	0.068	0.099
B3LYP/6-311G(3df,3pd)	0.229	0.241
B3LYP/6-31+G(d,p)	0.137	0.163
M062X/6-311++G(3df,3pd)	0.030	0.048
M062X/6-311+G(3df,3pd)	0.031	0.052
M062X/6-311G(3df,3pd)	0.272	0.316
M062X/6-31+G(d,p)	0.032	0.042

Table S8. Mean absolute deviation (MAD, eV) and maximum absolute deviation (MaxAD, eV) for adiabatic chemical hardness (η) relative to CBS-QB3 (averaged over all solvents).

Method	MAD	MaxAD
B3LYP/6-311++G(3df,3pd)	0.193	0.262
B3LYP/6-311+G(3df,3pd)	0.182	0.190
B3LYP/6-311G(3df,3pd)	0.108	0.111
B3LYP/6-31+G(d,p)	0.211	0.221
M062X/6-311++G(3df,3pd)	0.057	0.064
M062X/6-311+G(3df,3pd)	0.056	0.063
M062X/6-311G(3df,3pd)	0.009	0.027
M062X/6-31+G(d,p)	0.092	0.098

Table S9. Mean absolute deviation (MAD, eV^{-1}) and maximum absolute deviation (MaxAD, eV^{-1}) for adiabatic global softness (σ) relative to CBS-QB3 (averaged over all solvents).

Method	MAD	MaxAD
B3LYP/6-311++G(3df,3pd)	0.013	0.017
B3LYP/6-311+G(3df,3pd)	0.012	0.017
B3LYP/6-311G(3df,3pd)	0.007	0.010
B3LYP/6-31+G(d,p)	0.014	0.020
M062X/6-311++G(3df,3pd)	0.004	0.006
M062X/6-311+G(3df,3pd)	0.004	0.006
M062X/6-311G(3df,3pd)	0.000	0.001
M062X/6-31+G(d,p)	0.006	0.009

Table S10. Mean Absolute Deviation (MAD) and Maximum Absolute Deviation (MaxAD, EV) with respect to CBS-QB3 for electronegativity ($\chi = -\mu$) (averaged over all solvents)

Method	MAD	MaxAD
B3LYP/6-31+G(d,p)	0.073	0.079
B3LYP/6-311++G(3df,3pd)	0.103	0.122
B3LYP/6-311+G(3df,3pd)	0.114	0.122
B3LYP/6-311G(3df,3pd)	0.338	0.340
M062X/6-31+G(d,p)	0.060	0.074
M062X/6-311++G(3df,3pd)	0.087	0.093
M062X/6-311+G(3df,3pd)	0.088	0.095
M062X/6-311G(3df,3pd)	0.268	0.289

Table S11. Mean absolute deviation (MAD, eV) and maximum absolute deviation (MaxAD, eV) for adiabatic electrophilicity index (ω) relative to CBS-QB3 (averaged over all solvents).

Method	MAD	MaxAD
B3LYP/6-311++G(3df,3pd)	0.024	0.108
B3LYP/6-311+G(3df,3pd)	0.007	0.014
B3LYP/6-311G(3df,3pd)	0.329	0.363
B3LYP/6-31+G(d,p)	0.082	0.108
M062X/6-311++G(3df,3pd)	0.062	0.068
M062X/6-311+G(3df,3pd)	0.063	0.071
M062X/6-311G(3df,3pd)	0.323	0.354
M062X/6-31+G(d,p)	0.015	0.034

Table S12. Mean absolute deviation (MAD, eV) and maximum absolute deviation (MaxAD, eV) for adiabatic electroaccepting power (ω^{+}) relative to CBS-QB3 (averaged over all solvents).

Method	MAD	MaxAD
B3LYP/6-311++G(3df,3pd)	0.047	0.085
B3LYP/6-311+G(3df,3pd)	0.037	0.052
B3LYP/6-311G(3df,3pd)	0.174	0.208
B3LYP/6-31+G(d,p)	0.092	0.122
M062X/6-311++G(3df,3pd)	0.026	0.030
M062X/6-311+G(3df,3pd)	0.027	0.031
M062X/6-311G(3df,3pd)	0.189	0.224
M062X/6-31+G(d,p)	0.021	0.036

Table S13. Mean absolute deviation (MAD, eV) and maximum absolute deviation (MaxAD, eV) for adiabatic electrodonating power (ω^{-}) relative to CBS-QB3 (averaged over all solvents).

Method	MAD	MaxAD
B3LYP/6-311++G(3df,3pd)	0.074	0.095
B3LYP/6-311+G(3df,3pd)	0.077	0.096
B3LYP/6-311G(3df,3pd)	0.512	0.547
B3LYP/6-31+G(d,p)	0.022	0.044
M062X/6-311++G(3df,3pd)	0.113	0.120
M062X/6-311+G(3df,3pd)	0.114	0.124
M062X/6-311G(3df,3pd)	0.457	0.485
M062X/6-31+G(d,p)	0.041	0.081

Table S14. Mean absolute deviation (MAD) and maximum absolute deviation (MaxAD) for Gibbs solvation energies compared to CBS-QB3 (averaged over all solvents and species, in kcal/mol).

Method	MAD	MaxAD
B3LYP/6-311++G(3df,3pd)	1.250	3.450
B3LYP/6-311+G(3df,3pd)	1.240	3.420
B3LYP/6-311G(3df,3pd)	1.650	4.560
B3LYP/6-31+G(d,p)	1.850	5.120
M062X/6-311++G(3df,3pd)	0.850	2.340
M062X/6-311+G(3df,3pd)	0.840	2.320
M062X/6-311G(3df,3pd)	1.150	3.170
M062X/6-31+G(d,p)	0.750	2.070

Table S15. Mean absolute deviation (MAD, kcal/mol) and maximum absolute deviation (MaxAD, kcal/mol) for neutral solvation Gibbs energy relative to CBS-QB3 (averaged over all solvents).

Method	MAD	MaxAD
B3LYP/6-311++G(3df,3pd)	0.287	0.479
B3LYP/6-311+G(3df,3pd)	0.289	0.481
B3LYP/6-311G(3df,3pd)	0.524	0.784
B3LYP/6-31+G(d,p)	1.120	1.797
M062X/6-311++G(3df,3pd)	0.148	0.263
M062X/6-311+G(3df,3pd)	0.148	0.263
M062X/6-311G(3df,3pd)	0.489	0.740
M062X/6-31+G(d,p)	1.000	1.544

Table S16. Mean absolute deviation (MAD, kcal/mol) and maximum absolute deviation (MaxAD, kcal/mol) for cation solvation Gibbs energy relative to CBS-QB3 (averaged over all solvents).

Method	MAD	MaxAD
B3LYP/6-311++G(3df,3pd)	0.637	1.029
B3LYP/6-311+G(3df,3pd)	0.638	1.032
B3LYP/6-311G(3df,3pd)	0.270	0.643
B3LYP/6-31+G(d,p)	1.250	1.652
M062X/6-311++G(3df,3pd)	0.531	0.650
M062X/6-311+G(3df,3pd)	0.530	0.652
M062X/6-311G(3df,3pd)	0.280	0.531
M062X/6-31+G(d,p)	1.150	1.672

Table S17. Mean absolute deviation (MAD, kcal/mol) and maximum absolute deviation (MaxAD, kcal/mol) for anion solvation Gibbs energy relative to CBS-QB3 (averaged over all solvents).

Method	MAD	MaxAD
B3LYP/6-311++G(3df,3pd)	2.795	3.395
B3LYP/6-311+G(3df,3pd)	0.611	0.846
B3LYP/6-311G(3df,3pd)	0.402	0.608
B3LYP/6-31+G(d,p)	0.375	0.809
M062X/6-311++G(3df,3pd)	0.574	0.901
M062X/6-311+G(3df,3pd)	0.638	0.999
M062X/6-311G(3df,3pd)	0.487	0.711
M062X/6-31+G(d,p)	1.591	2.435

Table S18. Gibbs solvation energies (kcal/mol) for ascorbic acid species in water.

Method	Neutral	Cation	Anion
**CBS-QB3**	-10.928	-54.496	-58.306
B3LYP/6-31+G(d,p)	-12.725	-56.148	-59.115
B3LYP/6-311++G(3df,3pd)	-11.407	-55.243	-55.084
B3LYP/6-311+G(3df,3pd)	-11.409	-55.245	-57.653
B3LYP/6-311G(3df,3pd)	-10.144	-53.853	-57.731
M062X/6-31+G(d,p)	-12.472	-56.168	-60.741
M062X/6-311++G(3df,3pd)	-11.180	-55.114	-59.207
M062X/6-311+G(3df,3pd)	-11.180	-55.116	-59.305
M062X/6-311G(3df,3pd)	-10.188	-53.965	-59.017

Table S19. Gibbs solvation energies (kcal/mol) for ascorbic acid species in Benzene.

Method	Neutral	Cation	Anion
**CBS-QB3**	-4.8168	-29.0840	-30.7220
B3LYP/6-31+G(d,p)	-5.4750	-30.2680	-30.7260
B3LYP/6-311++G(3df,3pd)	-4.9705	-29.7090	-27.5140
B3LYP/6-311+G(3df,3pd)	-4.9711	-29.7110	-30.0350
B3LYP/6-311G(3df,3pd)	-4.4340	-29.0790	-31.0490
M062X/6-31+G(d,p)	-5.4499	-30.0080	-31.7730
M062X/6-311++G(3df,3pd)	-4.8513	-29.6860	-31.1000
M062X/6-311+G(3df,3pd)	-4.8513	-29.6770	-31.1450
M062X/6-311G(3df,3pd)	-4.4534	-29.1870	-31.1480

Table S20. Gibbs solvation energies (kcal/mol) for ascorbic acid species in Toluene.

Method	Neutral	Cation	Anion
**CBS-QB3**	-5.0295	-30.1840	-31.8470
B3LYP/6-31+G(d,p)	-5.7216	-31.3690	-31.8740
B3LYP/6-311++G(3df,3pd)	-5.1920	-30.8890	-28.6220
B3LYP/6-311+G(3df,3pd)	-5.1926	-30.8870	-31.1430
B3LYP/6-311G(3df,3pd)	-4.6342	-30.1650	-32.0020
M062X/6-31+G(d,p)	-5.6972	-31.1500	-32.9470
M062X/6-311++G(3df,3pd)	-5.0728	-30.7800	-32.2400
M062X/6-311+G(3df,3pd)	-5.0728	-30.7710	-32.2870
M062X/6-311G(3df,3pd)	-4.6567	-30.2330	-32.2830

Table S21. Gibbs solvation energies (kcal/mol) for ascorbic acid species in Chlorobenzene.

Method	Neutral	Cation	Anion
**CBS-QB3**	-8.1903	-43.5690	-47.2250
B3LYP/6-31+G(d,p)	-9.4459	-45.1190	-47.5500
B3LYP/6-311++G(3df,3pd)	-8.5071	-44.5980	-43.8300
B3LYP/6-311+G(3df,3pd)	-8.5097	-44.6010	-46.3790
B3LYP/6-311G(3df,3pd)	-7.5985	-43.5630	-46.6790
M062X/6-31+G(d,p)	-9.3549	-44.7270	-49.0970
M062X/6-311++G(3df,3pd)	-8.3703	-44.1910	-47.8470
M062X/6-311+G(3df,3pd)	-8.3716	-44.1940	-47.9160
M062X/6-311G(3df,3pd)	-7.6437	-43.2060	-47.7100

Table S22. Gibbs solvation energies (kcal/mol) for ascorbic acid species in Methanol.

Method	Neutral	Cation	Anion
**CBS-QB3**	-10.5940	-53.3530	-57.0580
B3LYP/6-31+G(d,p)	-12.3290	-54.9520	-57.8020
B3LYP/6-311++G(3df,3pd)	-11.0510	-54.0420	-53.8030
B3LYP/6-311+G(3df,3pd)	-11.0540	-54.0440	-56.3700
B3LYP/6-311G(3df,3pd)	-9.8312	-52.7380	-56.4540
M062X/6-31+G(d,p)	-12.1020	-55.0140	-59.4140
M062X/6-311++G(3df,3pd)	-10.8570	-53.9830	-57.9270
M062X/6-311+G(3df,3pd)	-10.8560	-53.9850	-58.0210
M062X/6-311G(3df,3pd)	-9.8877	-52.8730	-57.7380

Table S23. Gibbs solvation energies (kcal/mol) for ascorbic acid species in Ethanol.

Method	Neutral	Cation	Anion
**CBS-QB3**	-10.4170	-52.7380	-56.3840
B3LYP/6-31+G(d,p)	-12.1160	-54.3170	-57.1020
B3LYP/6-311++G(3df,3pd)	-10.8580	-53.4030	-53.1210
B3LYP/6-311+G(3df,3pd)	-10.8600	-53.4040	-55.6860
B3LYP/6-311G(3df,3pd)	-9.6649	-52.1340	-55.7760
M062X/6-31+G(d,p)	-11.8980	-54.4080	-58.7060
M062X/6-311++G(3df,3pd)	-10.6790	-53.3880	-57.2400
M062X/6-311+G(3df,3pd)	-10.6800	-53.3900	-57.3330
M062X/6-311G(3df,3pd)	-9.7226	-52.3040	-57.0550

Table S24. Adiabatic and vertical IP/EA (eV) in Benzene (ε = 2.27)

Method	IP adiab	IP vert	EA adiab	EA vert
M062X/6-31+G(d,p)	7.120	7.574	0.801	0.125
B3LYP/6-31+G(d,p)	6.986	7.370	0.917	0.398
B3LYP/6-311++G(3df,3pd)	6.980	7.370	0.850	0.518
M062X/6-311++G(3df,3pd)	7.131	7.595	0.740	0.089

Table S25. Adiabatic and vertical IP/EA (eV) in Toluene (ε = 2.38)

Method	IP adiab	IP vert	EA adiab	EA vert
M062X/6-31+G(d,p)	7.030	7.481	0.787	0.112
B3LYP/6-31+G(d,p)	6.896	7.278	0.902	0.373
B3LYP/6-311++G(3df,3pd)	6.889	7.278	0.835	0.488
M062X/6-311++G(3df,3pd)	7.041	7.502	0.727	0.074

Table S26. Adiabatic and vertical IP/EA (eV) in Chlorobenzene (ε = 5.62)

Method	IP adiab	IP vert	EA adiab	EA vert
M062X/6-31+G(d,p)	5.743	6.158	0.479	-0.178
B3LYP/6-31+G(d,p)	5.603	5.961	0.576	-0.025
B3LYP/6-311++G(3df,3pd)	5.586	5.954	0.506	-0.038
M062X/6-311++G(3df,3pd)	5.748	6.175	0.415	-0.230

Table S27. Adiabatic and vertical IP/EA (eV) in Methanol (ε = 32.61)

Method	IP adiab	IP vert	EA adiab	EA vert
M062X/6-31+G(d,p)	5.680	6.083	1.040	0.395
B3LYP/6-31+G(d,p)	5.551	5.890	1.126	0.532
B3LYP/6-311++G(3df,3pd)	5.533	5.882	1.057	0.480
M062X/6-311++G(3df,3pd)	5.686	6.100	0.976	0.343

Table S28. Adiabatic and vertical IP/EA (eV) in Ethanol (ε = 24.55)

Method	IP adiab	IP vert	EA adiab	EA vert
M062X/6-31+G(d,p)	5.762	6.166	1.081	0.436
B3LYP/6-31+G(d,p)	5.633	5.973	1.168	0.573
B3LYP/6-311++G(3df,3pd)	5.616	5.965	1.099	0.522
M062X/6-311++G(3df,3pd)	5.768	6.183	1.017	0.383

Table S29. Global reactivity descriptors for ascorbic acid computed using Koopmans theorem.

Descriptor	Vacuum	Benzene	Toluene	Chlorobenzene	Methanol	Ethanol	Water
**B3LYP/6-31+G(d,p)**							
IP (eV)	6.503	6.544	6.547	6.579	6.600	6.599	6.602
EA (eV)	1.167	1.205	1.208	1.241	1.262	1.261	1.265
E_g (eV)	5.337	5.339	5.339	5.338	5.338	5.338	5.337
η (eV)	2.668	2.669	2.669	2.669	2.669	2.669	2.669
σ (eV^{-1})	0.187	0.187	0.187	0.187	0.187	0.187	0.187
χ (eV)	3.835	3.875	3.877	3.910	3.931	3.930	3.933
ω (eV)	2.756	2.812	2.816	2.864	2.895	2.893	2.899
ω^- (eV)	5.007	5.083	5.088	5.153	5.194	5.192	5.199
ω^+ (eV)	1.172	1.209	1.211	1.243	1.263	1.262	1.266
**B3LYP/6-311++G(3df,3pd)**							
IP (eV)	6.523	6.560	6.562	6.591	6.609	6.607	6.610
EA (eV)	1.080	1.115	1.117	1.150	1.172	1.170	1.174
E_g (eV)	5.443	5.445	5.445	5.441	5.437	5.437	5.436
η (eV)	2.721	2.722	2.722	2.720	2.718	2.719	2.718
σ (eV^{-1})	0.184	0.184	0.184	0.184	0.184	0.184	0.184
χ (eV)	3.802	3.837	3.839	3.871	3.890	3.889	3.892
ω (eV)	2.655	2.704	2.707	2.754	2.783	2.781	2.787
ω^- (eV)	4.896	4.963	4.967	5.029	5.068	5.065	5.073
ω^+ (eV)	1.095	1.126	1.128	1.158	1.178	1.177	1.181
**B3LYP/6-311+G(3df,3pd)**							
IP (eV)	6.523	6.559	6.561	6.591	6.609	6.607	6.610
EA (eV)	1.072	1.112	1.114	1.148	1.170	1.169	1.173
E_g (eV)	5.450	5.447	5.447	5.443	5.438	5.438	5.437
η (eV)	2.725	2.724	2.724	2.721	2.719	2.719	2.719
σ (eV^{-1})	0.183	0.184	0.184	0.184	0.184	0.184	0.184
χ (eV)	3.798	3.836	3.838	3.870	3.889	3.888	3.892
ω (eV)	2.646	2.701	2.704	2.751	2.781	2.780	2.785
ω^- (eV)	4.885	4.959	4.963	5.026	5.066	5.063	5.071
ω^+ (eV)	1.088	1.124	1.126	1.157	1.177	1.175	1.179
**B3LYP/6-311G(3df,3pd)**							
IP (eV)	6.317	6.363	6.365	6.398	6.418	6.417	6.420
EA (eV)	0.832	0.884	0.886	0.924	0.947	0.946	0.950
E_g (eV)	5.485	5.479	5.479	5.474	5.471	5.471	5.470
η (eV)	2.742	2.740	2.739	2.737	2.735	2.735	2.735
σ (eV^{-1})	0.182	0.183	0.183	0.183	0.183	0.183	0.183
χ (eV)	3.575	3.623	3.626	3.661	3.683	3.681	3.685
ω (eV)	2.330	2.396	2.399	2.448	2.479	2.477	2.483
ω^- (eV)	4.460	4.550	4.555	4.620	4.662	4.660	4.667
ω^+ (eV)	0.885	0.927	0.929	0.960	0.980	0.978	0.982
**HF/6-311++G(3df,3pd)**							
IP (eV)	9.463	9.503	9.506	—	9.548	9.547	9.550
EA (eV)	-0.825	-1.027	-1.034	—	-1.178	-1.175	-1.183
E_g (eV)	10.288	10.530	10.540	—	10.725	10.722	10.733
η (eV)	5.144	5.265	5.270	—	5.363	5.361	5.366
σ (eV^{-1})	0.097	0.095	0.095	—	0.093	0.093	0.093
χ (eV)	4.319	4.238	4.236	—	4.185	4.186	4.183
ω (eV)	1.813	1.706	1.702	—	1.633	1.635	1.630
ω^- (eV)	4.616	4.483	4.479	—	4.396	4.398	4.393
ω^+ (eV)	0.297	0.245	0.243	—	0.211	0.211	0.210
**HF/6-311+G(3df,3pd)**							
IP (eV)	9.463	9.504	9.506	—	9.548	9.548	9.550
EA (eV)	-1.425	-1.634	-1.641	—	-1.717	-1.717	-1.718
E_g (eV)	10.888	11.138	11.147	—	11.265	11.265	11.267
η (eV)	5.444	5.569	5.573	—	5.633	5.632	5.634
σ (eV^{-1})	0.092	0.090	0.090	—	0.089	0.089	0.089
χ (eV)	4.019	3.935	3.933	—	3.915	3.915	3.916
ω (eV)	1.484	1.390	1.388	—	1.361	1.361	1.361
ω^- (eV)	4.174	4.053	4.051	—	4.023	4.023	4.023
ω^+ (eV)	0.155	0.119	0.118	—	0.107	0.107	0.107
**HF/6-311G(3df,3pd)**							
IP (eV)	9.338	9.384	9.387	—	9.432	9.431	9.434
EA (eV)	-3.061	-3.347	-3.356	—	-3.469	-3.468	-3.471
E_g (eV)	12.398	12.731	12.743	—	12.901	12.899	12.904
η (eV)	6.199	6.365	6.371	—	6.450	6.450	6.452
σ (eV^{-1})	0.081	0.079	0.078	—	0.078	0.078	0.077
χ (eV)	3.138	3.019	3.015	—	2.981	2.982	2.981
ω (eV)	0.794	0.716	0.713	—	0.689	0.689	0.689
ω^- (eV)	3.139	3.021	3.017	—	2.986	2.986	2.986
ω^+ (eV)	0.000	0.002	0.002	—	0.005	0.005	0.005

Table S30. Global reactivity descriptors for ascorbic acid computed using Koopmans theorem (continued).

Descriptor	Vacuum	Benzene	Toluene	Chlorobenzene	Methanol	Ethanol	Water
**M062X/6-311++G(3df,3pd)**							
IP (eV)	7.959	8.004	8.005	8.039	8.060	8.059	8.062
EA (eV)	0.508	0.281	0.272	0.176	0.132	0.134	0.128
E_g (eV)	7.450	7.723	7.733	7.863	7.928	7.924	7.935
η (eV)	3.725	3.862	3.866	3.932	3.964	3.962	3.967
σ (eV^{-1})	0.134	0.129	0.129	0.127	0.126	0.126	0.126
χ (eV)	4.233	4.142	4.139	4.107	4.096	4.097	4.095
ω (eV)	2.405	2.222	2.215	2.145	2.116	2.118	2.114
ω^- (eV)	4.988	4.775	4.768	4.690	4.660	4.661	4.657
ω^+ (eV)	0.754	0.633	0.629	0.583	0.564	0.565	0.562
**M062X/6-311+G(3df,3pd)**							
IP (eV)	7.959	8.004	8.005	8.039	8.060	8.059	8.062
EA (eV)	0.252	0.035	0.030	0.004	0.012	0.011	0.013
E_g (eV)	7.707	7.969	7.975	8.034	8.048	8.048	8.049
η (eV)	3.853	3.984	3.988	4.017	4.024	4.024	4.025
σ (eV^{-1})	0.130	0.125	0.125	0.124	0.124	0.124	0.124
χ (eV)	4.105	4.019	4.018	4.022	4.036	4.035	4.038
ω (eV)	2.187	2.027	2.024	2.013	2.024	2.023	2.026
ω^- (eV)	4.721	4.535	4.531	4.526	4.545	4.543	4.548
ω^+ (eV)	0.616	0.516	0.514	0.504	0.509	0.508	0.510
**M062X/6-311G(3df,3pd)**							
IP (eV)	7.799	7.853	7.855	7.891	7.914	7.913	7.918
EA (eV)	-0.347	-0.291	-0.288	-0.249	-0.223	-0.224	-0.220
E_g (eV)	8.146	8.144	8.143	8.141	8.137	8.138	8.138
η (eV)	4.073	4.072	4.072	4.070	4.069	4.069	4.069
σ (eV^{-1})	0.123	0.123	0.123	0.123	0.123	0.123	0.123
χ (eV)	3.726	3.781	3.783	3.821	3.846	3.844	3.849
ω (eV)	1.704	1.755	1.758	1.794	1.817	1.816	1.821
ω^- (eV)	4.076	4.155	4.158	4.213	4.249	4.247	4.254
ω^+ (eV)	0.350	0.374	0.375	0.392	0.403	0.403	0.405
